# 

**Published:** 1911-07

**Authors:** 


					﻿PUBLISHED MONTHLY.	ATLANTA	SUBSCRIPTION $1.00
JOURNAL RECORD
OF MEDlClNl^RV
I AuGAlO. 1311
Successor A AtlanU Me‘,lcal and Sur«ical Journal’	AWtrYpar
(and Southern Medical Record, Established	j
Vol. LVII.	Atlanta, Ga., July, 1911.	No. 4
				

